# Ischemia-Reperfusion Injury of the Cochlea: Pharmacological Strategies for Cochlear Protection and Implications of Glutamate and Reactive Oxygen Species

**DOI:** 10.2174/157015910791233123

**Published:** 2010-06

**Authors:** Keiji Tabuchi, Bungo Nishimura, Shuho Tanaka, Kentaro Hayashi, Yuki Hirose, Akira Hara

**Affiliations:** Department of Otolaryngology, Graduate School of Comprehensive Human Sciences, University of Tsukuba, Tsukuba, Japan

**Keywords:** Cochlea, Blood flow, Ischemia-reperfusion injury, Excitotoxicity, Oxidative damage.

## Abstract

A large amount of energy produced by active aerobic metabolism is necessary for the cochlea to maintain its function. This makes the cochlea vulnerable to blockade of cochlear blood flow and interruption of the oxygen supply. Although certain forms of human idiopathic sudden sensorineural hearing loss reportedly arise from ischemic injury, the pathological mechanism of cochlear ischemia-reperfusion injury has not been fully elucidated. Recent animal studies have shed light on the mechanisms of cochlear ischemia-reperfusion injury. It will help in the understanding of the pathology of cochlear ischemia-reperfusion injury to classify this injury into ischemic injury and reperfusion injury. Excitotoxicity, mainly observed during the ischemic period, aggravates the injury of primary auditory neurons. On the other hand, oxidative damage induced by hydroxyl radicals and nitric oxide enhances cochlear reperfusion injury. This article briefly summarizes the generation mechanisms of cochlear ischemia-reperfusion injury and potential therapeutic targets that could be developed for the effective management of this injury type.

## INTRODUCTION

The sole purpose of the circulatory system is transportation. It performs oxygen and nutrient delivery and metabolic by-product removal. The labyrinthine artery is a terminal artery for cochlear blood supply. The cochlea is highly dependent on blood and oxygen supply to maintain its function. In animal models of cochlear ischemia, the cochlea becomes hypoxic or ischemic within one minute after occlusion of the labyrinthine artery.

It has been proposed that one of the major causes of human idiopathic sudden sensorineural hearing loss is impaired blood flow and oxygen delivery to the cochlea [1]. The aim of this review is to provide an overview of recent insights regarding the pathogenesis of cochlear ischemia-reperfusion injury observed in animal studies.

## CHANGES IN COCHLEAR POTENTIALS AND MORPHOLOGIES DURING ISCHEMIA

Adenosine triphosphate (ATP), a critical compound in cellular energy metabolism, is synthesized in mitochondria from adenosine diphosphate (ADP) and inorganic phosphate in the presence of oxygen and nicotinamide adenine dinucleotide (NADH). Interruption of the blood supply causes ATP depletion in the cochlea [2]. If this ATP depletion during ischemia is severe, it may lead to the compromised function of all cochlear cells. Perlman *et al*. [3] were the first to perform an extensive study of functional and histological alterations of the cochlea during and after reversible cochlear ischemia in the guinea pig. Because the stria vascularis requires high-level energy supply to maintain endocochlear potential (EP) levels, EP rapidly declines immediately after the onset of ischemia to reach its lowest level (-30 to -40 mV). EP is essential to maintain the function of hair cells and cochlear afferent neurons, and its decrease results in the subsequent decrease of other cochlear potentials, such as cochlear microphonic (CM), distortion-product otoacoustic emission (DPOAE), and compound action potential (CAP) [4].

Generally, interruption of the blood supply causes various morphological changes including cellular, mitochondrial, and nuclear swelling, swelling of the endoplasmic reticulum, clearing of the cytoplasm, bleb formation, and the condensation of chromatin. However, the tolerance of cochlear cells to ischemia is variable among cell types. Dendrites of cochlear afferent neurons are one of the components most vulnerable to ischemia. The swelling of afferent neurons is observed in cases of cochlear ischemia of 5 minutes or longer. Ischemia-induced morphological changes are also observed in hair cells. Approximately 15 and 30 minutes of ischemia causes the swelling of outer and inner hair cells, respectively. As the ischemic period prolongs, more severe morphological changes are observed, such as the swelling of nuclei, bleb formation, and rupture of the cell membrane. Several hours of ischemia induces disintegration of the organ of Corti [4].

## EXCITOTOXICITY OF COCHLEAR AFFERENT NEURONS DURING THE ISCHEMIC PERIOD

Excitotoxicity is an important and well-accepted theory proposed by Olney *et al*. [5], which explains one of the major pathophysiologies of brain ischemia. Excitotoxicity is caused by excessive amounts of glutamate in synaptic cleft. Glutamate is a putative excitatory neurotransmitter between inner hair cells and afferent neurons [6, 7]. Although vital for cochlear function, excessively released glutamate and the failure of its removal from synaptic clefts can lead to the excitotoxicity of cochlear afferent neurons due to the loss of ionic homeostasis. Puel *et al*. [8] showed that cochlear ischemia causes the excitotoxicity of afferent neurons to induce the massive swelling of afferent dendrites.

The excessive efflux of glutamate from inner hair cells results in cochlear excitotoxicity. Massive glutamate efflux in the perilymph of the cochlea is observed during cochlear ischemia [9, 10]. Besides this excessive efflux, the failed uptake of glutamate from the synaptic clefts into surrounding cells also causes cochlear excitotoxicity. To date, five subtypes of Na^+^-dependent glutamate transporter: GLT-1, GLAST, EAAC1, EAAT4, and EAAT5, have been cloned [11-13]. Previous studies have demonstrated that GLAST is present in the supporting cells surrounding inner hair cells and in the satellite cells surrounding spiral ganglion neurons in the rodent cochlea [14, 15]. Takumi *et al*. [16] demonstrated the colocalization of GLAST and glutamine synthetase in supporting cells around inner hair cells. Based on their findings, it is suggested that glutamate released from the presynaptic region is taken into adjacent supporting cells using GLAST and then converted to glutamine in the supporting cells [17]. Glutamate transporters are required to terminate glutamate actions. Hakuba *et al*. [18] has shown that GLAST knockout mice are vulnerable to cochlear excitotoxicity. Although GLT-1 and EAAC1 also exist in the cochlea, the involvement of these glutamate transporters in cochlear exctitotoxicity has not yet been clarified.

It is now widely accepted that glutamate receptors fall into two major classes: the ionotropic receptors formed by ligand-gated cation channels and the metabotropic receptors that are coupled to G-proteins and act though intracellular chemical mechanisms [19-21]. Eight subtypes of metabotropic glutamate receptors have been currently identified, and subdivided into three groups according to sequence homology and response to agonists [22]. Ionotropic glutamate receptors are divided into three types: AMPA, kainite, and N-methyl-D-aspartate (NMDA) receptors. The swelling of afferent dendrites is induced via non-NMDA ionotropic glutamate receptors in the cochlea because the application of APMA and kainic acid to the cochlea leads to the same swelling of afferent dendrites. An AMPA/kainate receptor antagonist, 6-7-dinitroquinoxaline-2,3-dione (DNQX), protected most of the radial dendrites from the ischemia-induced swelling [8]. In contrast to the involvement of AMPA/kainate receptors in the cochlear excitotoxicity and cochlear ischemic injury, a definitive role of metabotropic and NMDA receptors in cochlear ischemic injury has not been shown. MK-801 and D-2-amino-5-phosphonopentanoate, NMDA receptor antagonists, did not show protective effects against cochlear ischemia [5, 23].

It is now considered that the excitotoxicity of cochlear afferent neurons often induces temporary hearing threshold shifts [24]. However, Sun *et al*. [25] demonstrated that some cochlear excitotoxicity might induce irreversible cochlear lesions because the number of cochlear ganglion neurons decreased after exposure to high concentration of kainic acid.

**Fig. (1):**
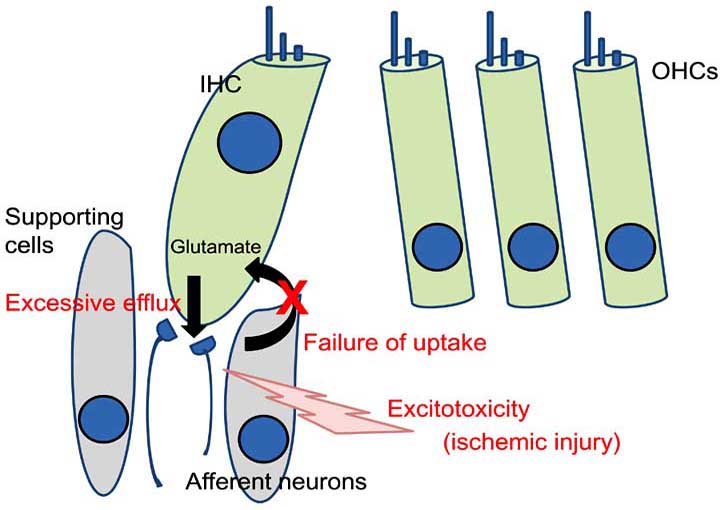
Schematic view of cochlear ischemic injury. Glutamate is a putative neurotransmitter between inner hair cells and afferent neurons. Glutamate released from inner hair cells is promptly taken into the inner hair cells and supporting cells by glutamate transporters in the normal cochlea. Ischemia induces the excessive efflux of glutamate and dysfunction of the glutamate transporter. These cause the excitotoxicity of afferent neurons. IHC: inner hair cell. OHCs: outer hair cells.

The swelling of afferent neurons is observed in cases of cochlear ischemia of 5 minutes or longer. Excitotoxicity is undoubtedly one of the leading causes of cochlear afferent neuron injury during ischemia (Fig. **1**). 

## CHARACTERISTICS OF COCHLEAR INJURY DURING THE REPERFUSION PERIOD

Although the cochlear response to ischemia has been extensively studied, the effects of reperfusion on the cochlea have recently received new attention. Recent animal models of cochlear ischemic injury are suitable to examine the time course of cochlear functions during reperfusion [9, 26-29]. The CAP threshold representing the function of cochlear afferent neurons gradually improved during the reperfusion period. On the other hand, DPOAE (representing the function of outer hair cells) initially recovered with time until 20 minutes after the onset of reperfusion when the cochlear circulation was restored. Thereafter the amplitude of DPOAE gradually decreased toward the noise level, whereas CAP gradually improved during this time period, as mentioned above. CM, another indicator of hair cell function, exhibited essentially the same time course during the reperfusion period as DPOAE [4]. Based on these findings, it is considered that the main lesion of cochlear reperfusion injury is in outer hair cells. In morphological studies of the cochlea, reperfusion of the cochlea induces swelling of the outer hair cells and eventual rupture of the outer hair cell membrane and nucleus [4]. The swelling of terminals of afferent dendrites improved during the reperfusion period [4]. This morphological finding is in agreement with the fact that CAP gradually improved whereas DPOAE decreased during reperfusion. Excitotoxicity during ischemia is involved in the swelling of afferent neurons, which improved during reperfusion. It has been shown that the glutamate concentration in the perilymph, which increased during the ischemic period, decreased toward the pre-ischemia level upon reperfusion [10].

## INVOLVEMENT OF REACTIVE OXYGEN SPECIES IN REPERFUSION INJURY OF THE COCHLEA

As described in a previous section, outer hair cells functionally and structurally deteriorate during the early reperfusion period. Recent studies have indicated that free radicals such as hydroxyl radicals and nitric oxide (NO) are involved in the injury of outer hair cells.

It has been generally accepted that reoxygenation causes the accumulation of superoxide anions in tissues. The superoxide anion is converted by superoxide dismutase to hydrogen peroxide, which is relatively unreactive toward most organic molecules. However, the hydroxyl radical, a potent oxidant capable of promoting oxidative damage, may be generated by the reaction of hydrogen peroxide with iron (Fenton reaction). Hydroxyl radicals can oxidize DNA, proteins, and structural polymers including hyaluronic acid. In addition, they induce the peroxidation of polyunsaturated fatty acid, which damages biological membranes [30]. Hypoxia increased the iron concentration in the perilymph of the cochlea [31], and the marked production of hydroxyl radicals was demonstrated during the re-oxygenation phase after hypoxia [32]. These findings suggest that hydroxyl radicals were generated *via *the Fenton reaction during the early reperfusion period in cochlear ischemia-reperfusion injury.

Generally, the main source of superoxide anions during reperfusion is considered to be mitochondria. Reduced intermediates of mitochondrial electron transport systems accumulate during ischemia and are oxidized during reperfusion (by reoxygenation), leading to the production of superoxide anions. Another potential source of superoxide anions is xanthine oxidase, which oxidizes hypoxanthine to xanthine and xanthine to urate. However, xanthine oxidase may not be involved in the generation of free radicals because allopurinol and oxypurinol, potent inhibitors of xanthine oxidase, did not exhibit any effect on cochlear ischemia-reperfusion injury [33].

**Fig. (2):**
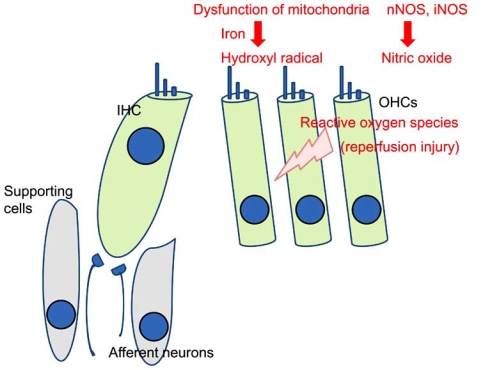
Schematic view of cochlear reperfusion injury. Reoxygenation leads to the generation of superoxide anions, in which mitochondrial dysfunction may be involved. Superoxide anions are converted into hydroxyl radicals via the Fenton reaction in the presence of iron. NO is also generated by nNOS and iNOS during the reperfusion period. These reactive oxygen species cause the reperfusion injury of outer hair cells. IHC: inner hair cell. OHCs: outer hair cells.

Nitric oxide (NO) is also involved in the generation mechanism of cochlear ischemia-reperfusion injury. Transient ischemia causes a marked increase of NO production in the perilymph in the reperfusion period [34]. To date, three isoforms of NO synthase (NOS) have been distinguished: neuronal NOS (nNOS), endothelial NOS (eNOS), and inducible NOS (iNOS). The constitutive isoforms of NOS (i.e., nNOS and eNOS), but not iNOS are expressed in the normal cochlea [35, 36]. Recent findings suggest that NO generated by nNOS and iNOS deteriorates the cochlea in ischemia-reperfusion injury [29, 37, 38]. NO synthesized by iNOS plays a deleterious role in cerebral ischemia, and ischemia-induced iNOS mRNA expression and iNOS activity were delayed by several hours (more than 6) after transient cerebral ischemia [39, 40]. The administration of aminoguanidine, an iNOS inhibitor, decreased the postischemic shift of the ABR or CAP threshold at 1 to 5 days after 60-minute ischemia, but not at 4 hours after ischemia [29, 38]. These results strongly suggest that NO generated by nNOS and by iNOS aggravates cochlear injury in early and late phases of reperfusion, respectively (Fig. **2**). 

## TIME THRESHOLD OF ISCHEMIA TO INDUCE COCHLEAR DYSFUNCTION

Individual tissues have quite variable oxygen requirements. For example, the brain cannot tolerate hypoxia for more than a few minutes without permanent injury, although skeletal muscles can tolerate severe hypoxia or ischemia for several hours [41]. Several researchers have examined the time threshold of ischemia to induce cochlear dysfunction using animal models. EP and other cochlear potentials start to decrease within a minute after the cochlea is subjected to ischemia. When circulation is resumed, cochlear functions fully recover if the ischemic period is less than 10 minutes. However, DPOAE significantly decreases after ischemia of 10 minutes compared with the preischemic level [26]. CAP thresholds also elevate after ischemia of 10 minutes or longer [28]. These findings support the idea that ischemia of 10 minutes or longer at least transiently elevates the hearing threshold. When cochlear functions are followed for long periods after the onset of recirculation, ischemia of 15 to 30 minutes duration induces hair cell loss and permanent cochlear dysfunction [9, 29].

## PHARMACOLOGICAL STRATEGIES TO PROTECT THE COCHLEA AGAINST ISCHEMIA-REPERFUSION INJURY

This section presents a brief overview of experimental data concerning pharmacological strategies for cochlear protection against ischemia-reperfusion injury.

### 1. Excitotoxicity

An AMPA/kainate receptor antagonist, 6-7-dinitro-quinoxaline-2,3-dione (DNQX), protected most radial dendrites from ischemia-induced swelling [8]. In contrast to the protective effect of AMPA/kainate receptor inhibitors against cochlear excitotoxicity and cochlear ischemic injury, specific NMDA receptor antagonists including MK-801 and D-2-amino-5-phosphonopentanoate did not show any protective effect against cochlear ischemia [8, 23].

There are several agents reported to reduce cochlear excitotoxicity including a GABA(A) agonist (muscimol [24]), dopamine [42, 43], riluzole [44], ebselen [45], and pituitary adenyl cyclase-activating polypeptide (PACAP) [46]. Because the excitotoxicity of cochlear afferent neurons often induces temporary hearing threshold shifts [24], these agents are potential therapeutic targets to prevent temporary hearing deterioration.

The majority of clinical trials of glutamatergic antagonists were conducted in the fields of stroke, traumatic brain injury and dementia. However, treatment with glutamatergic antagonists failed to obtain even modest beneficial results, and those clinical trials were interrupted [47]. In addition, the adverse effect profile of glutamatergic antagonists has been described. Based on the findings obtained in central nervous system disorders, it seems reasonable to consider local application of glutamatergic antagonists for prevention of temporary elevation of hearing thresholds.

### 2. Free Radical Scavengers and NOS Inhibitors

Seidman and Quirk [48] first demonstrated the involvement of free radicals in cochlear ischemia-reperfusion injury. Since their report, several free radical scavengers have been reported to protect against cochlear ischemia-reperfusion injury, including U74006F, mannitol, edaravone, and dimethylthiourea [4, 28, 48, 49].

It has been suggested that superoxide and hydrogen peroxide have a limited reactivity with most biological molecules *in vivo*, and their interaction in the presence of a transition metal (e.g., iron) forms far more reactive hydroxyl radicals by the iron-catalyzed Haber-Weiss (Fenton) reaction that contribute to tissue injury [30]. The protective effects of deferoxamine, an iron chelator, in cochlear ischemia have been established [38, 50].

In addition to free radical scavengers and iron chletors, NOS inhibitors protect the cochlea. N-nitro-L-arginine decreased the postischemic CAP threshold shift [28]. Inhibitors of nNOS (7-nitroindazole, 3-bromo-7-nitroindazole) and iNOS (aminoguanidine) also protect the cochlea against ischemia-reperfusion injury [29, 38, 50].

Outer hair cells deteriorate during reperfusion, and cochlear reperfusion injury is most evidently observed in outer hair cells. DPOAE studies demonstrated that free radical scavengers, iron chelators, and NOS inhibitors protected outer hair cells from reperfusion injury [4, 50].

### 3. Adenosine Receptor Agonists

Although information on the cochlea is limited, several mechanisms have been proposed to account for the protective effects of A1 adenosine receptor agonists in nervous tissues. It is thought that deleterious Ca^2+^ influx due to glutamate excitotoxicity is a primary target for neuroprotective adenosine actions in the central nervous system [51-53]. The excitotoxicity of cochlear afferent dendrites induced by kainic acid was attenuated by the application of 2-chloro-N6-cyclopentyladenosine (CCPA), a highly selective adenosine A1 receptor agonist. Ford *et al*. [54] reported the effects of the round window application of R-PIA, a selective adenosine A1 receptor agonist, on cochlear antioxidant enzymes, such as superoxide dismutase, glutathione peroxidase, and catalase. R-PIA elicited significant increases in the activity of these antioxidant enzymes and significantly reduced the levels of malondialdehyde, a marker of lipid peroxidation, generated in the cochlea. Namely, it is possible that adenosine A1 receptor agonists protect the cochlea against ischemia-reperfusion injury by attenuating glutamate excitotoxicity and through a radical scavenging effect. Indeed, CCPA attenuated cochlear injury induced by transient ischemia [55].

### 4. Growth Factors

Several authors have shown that growth factors like insulin-like growth factor-1 (IGF-1), epidermal growth factor (EGF), and fibroblast growth factor may protect hair cells against damage triggered by ototoxic drugs or aging. These growth factors are important for the normal development and survival of cells including hair cells of the organ of Corti [56, 57]. Although the effects of growth factors on cochlear ischemia-reperfusion injury have not been fully elucidated, the protective effects of erythropoietin and IGF-1 have been reported [58, 59].

### 5. Steroids

Glucocorticoids have been widely used in the treatment of idiopathic sudden sensorineural hearing loss in humans. In a basic animal study, the cochlear function 4 hours after transient ischemia was significantly improved by glucocorticoids, prednisolone and methylprednisolone, at a relatively wide dose range in the case of pre-ischemic administration. On post-ischemic administration, higher doses of glucocorticoids were necessary to ameliorate cochlear ischemia-reperfusion injury [54]. It is considered that glucocorticoids do not promote cochlear blood flow to protect hair cells [60, 61], but directly protect outer hair cells [62].

Dehydroepiandrosterone reportedly exhibits protective effects against cochlear ischemia-reperfusion injury [63]. Interestingly, dehydroepiandrosterone also protects hair cells against acoustic injury [64], suggesting that this steroid protects hair cells and the cochlea against other forms of cochlear injury.

## CONCLUSION

An understanding of the mechanisms of cochlear ischemia-reperfusion injury is mandatory for the development of therapeutic interventions directed toward this type of injury. When organs are rendered ischemic, there is a shift from aerobic to anaerobic metabolism, which results in a decrease in cellular ATP. Excitotoxicity induced by glutamate efflux and its failed removal from extracellular spaces during ischemia aggravates the ischemic injury of primary auditory neurons. Outer hair cells are the most vulnerable to cochlear reperfusion injury. Reactive oxygen species including hydroxyl radicals and NO are leading factors in the reperfusion injury of outer hair cells. Once recirculation is achieved after ischemia, efforts are needed to prevent cochlear injury induced by reactive oxygen species and to minimize the loss of cochlear function. Many chemical agents including glutamate receptor antagonists, free radical scavengers and NOS inhibitors, adenosine receptor agonists, and growth factors are under investigation. Further research will clarify the details of the pathogenesis of cochlear ischemia-reperfusion injuries, and efforts should be made to establish strategies to protect the cochlea.
